# Anarchic centromeres: deciphering order from apparent chaos^☆^

**DOI:** 10.1016/j.ceb.2013.09.004

**Published:** 2014-02

**Authors:** Sandra Catania, Robin C Allshire

**Affiliations:** Wellcome Trust Centre for Cell Biology, School of Biological Sciences, The University of Edinburgh, 6.34 Swann Building, Mayfield Road, Edinburgh EH9 3JR, Scotland, UK

## Abstract

Specialised chromatin in which canonical histone H3 is replaced by CENP-A, an H3 related protein, is a signature of active centromeres and provides the foundation for kinetochore assembly. The location of centromeres is not fixed since centromeres can be inactivated and new centromeres can arise at novel locations independently of specific DNA sequence elements. Therefore, the establishment and maintenance of CENP-A chromatin and kinetochores provide an exquisite example of genuine epigenetic regulation. The composition of CENP-A nucleosomes is contentious but several studies suggest that, like regular H3 particles, they are octamers. Recent analyses have provided insight into how CENP-A is recognised and propagated, identified roles for post-translational modifications and dissected how CENP-A recruits other centromere proteins to mediate kinetochore assembly.

**Current Opinion in Cell Biology** 2014, **26**:41–50

This review comes from a themed issue on **Cell architecture**

Edited by **Sue Biggins** and **Matthew D Welch**

For a complete overview see the Issue and the Editorial

Available online 19th October 2013

0955-0674/$ – see front matter, © 2013 The Authors. Published by Elsevier Ltd. This is an open access article under the CC BY license (http://creativecommons.org/licenses/by/3.0/).

**http://dx.doi.org/10.1016/j.ceb.2013.09.004**

“Chaos is merely order waiting to be deciphered” — José de Sousa Saramago

## Introduction

Centromeres are the sites at which the machinery, collectively known as the kinetochore, required to accurately segregate chromosomes is assembled. Following replication, the resulting sister-kinetochores on each sister-chromatid ensure that one chromatid from each chromosome is transmitted to each daughter nucleus. Kinetochores are an amalgamation of integrated functional modules: they include devices which ensure that sister-chromatids remain associated at centromeres (cohesion) [1], and sensors (the spindle assembly checkpoint) for detecting when all sister-kinetochores have attached to microtubules anchored at opposite spindle poles (bi-orientation). Once all sister-kinetochores are bi-oriented, this sensor throws a switch allowing the release of sister-centromeres and their separation into two new nuclei [2–4]. This separation and movement to opposite poles are mediated by the attachment of each kinetochore to microtubules utilising another apparatus that binds directly to microtubules [1,4–6].

The integration of these modules into a single unit allows the presence of an unattached kinetochore to be sensed and transduced via a signalling cascade that ultimately prevents the release of the tethers between all sister-centromeres and thereby halts the movement of chromosomes and the completion of both nuclear and cellular division until the problem is resolved. Gain or loss of chromosomes (aneuploidy) is one step on the path to forming cancerous cells [2–4,7]. Kinetochores therefore provide exquisite accuracy to the process of chromosome segregation so that over the course of thousands of cell division few detrimental chromosome segregation errors occur.

This view of the kinetochore as a highly honed and accurate piece of cellular engineering seems incompatible with the seemingly haphazard processes that in many organisms govern where kinetochores are assembled on chromosomes. Each chromosome must only assemble a single kinetochore, chromosomes with two kinetochores are intrinsically unstable (an exception being holocentric chromosomes). An effective way to ensure the assembly of only one kinetochore per chromosome would be to couple kinetochore assembly to a unique DNA sequence. Indeed, at budding yeast (*Saccharomyces cerevisiae*) centromeres, which have contributed greatly to our knowledge of centromere–kinetochore structure and function, specific centromere proteins bind to a DNA sequence motif which in turn ensures kinetochore assembly at that location [8]. Single base changes in a key centromere DNA element, or mutation of its DNA binding proteins, obliterate kinetochore assembly. This seems a completely logical template for centromere specification. However, in organisms with complex regional centromeres, the processes involved in centromere placement appear much more anarchic. In all organisms, the main driver of centromere specification appears to be the assembly of specialised chromatin containing the histone H3 variant generally known as CENP-A and called as CID (*Drosophila*), Cse4 (*S. cerevisiae*) and Cnp1 (*Schizosaccharomyces pombe*). The structure of CENP-A nucleosomes, their post-translational modifications (PTMs), the timing and mechanism of their deposition may all influence where kinetochores are assembled. Here we discuss recent developments that contribute to our understanding of CENP-A chromatin in addition to where and how it seeds kinetochore assembly.

## Inducing new centromere formation

The inactivation of one centromere on dicentric chromosomes and the appearance of new centromeres at novel locations support the view that the establishment and propagation of centromeres are epigenetically regulated.

**Figure 1:**
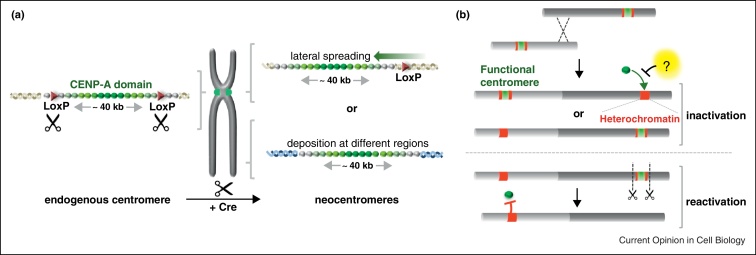
Establishment and propagation of centromeres are epigenetically regulated. **(a)** Centromere repositioning results in neocentromere formation at ectopic loci. In DT-40 cells, Cre-induced deletion of the centromere from chromosome Z generated surviving colonies able to retain chromosome Z and possessing neocentromeres. The majority of the characterised neocentromeres arose in region flanking the original chromosome Z suggesting that a low level of CENP-A may function to seed neocentromere formation. The same lateral spreading was also described in *Candida*. The remaining neocentromeres are localised on different regions of chromosome Z, suggesting that the original centromere is not unique in being able to attract CENP-A and assemble kinetochores. At each neocentromere, CENP-A occupies a ∼40 kb region, similar in size to original centromere. **(b)** In fission yeast, forced fusion of non-homologous chromosomes leads to formation of a dicentric chromosome. In some surviving cells, dicentric chromosomes are converted into monocentric chromosomes by deletion of one of the centromeres. In survivors in which DNA rearrangements have not occurred, the one of the centromeres has become inactivated and is coated in H3K9me-dependent heterochromatin. Although heterochromatin is associated with the inactive centromere, it is not required for centromere inactivation and other unknown mechanisms may be involved. However, heterochromatin does prevent reactivation of the inactive centromere, leading to neocentromere formation when the functional centromere on a dicentric is deleted.

Regional centromeres in most organisms are restricted to the same single locus on each chromosome. These regions usually contain arrays of repetitive elements, such as alpha satellite repeat arrays at human centromeres, with divergent but related repeats found at each centromere [9], which may represent a preferred substrate directed by DNA binding factors (e.g. CENP-B) [10]. Thus DNA elements are involved in genetically specifying centromere placement; however, some regional centromeres are associated with unique DNA sequences. The lack of obvious common features suggests that epigenetic mechanisms direct formation of these centromeres. For example, new centromeres are formed on regions of horse, orang-utan and potato chromosomes that contain no satellite repeats [11–13]. Analyses of CENP-A distribution in chicken DT40 cells have shown that two of the 10 macro-chromosomes (Chr 5 and Z) lack repetitive DNA and display single CENP-A peaks that occupy ∼30 kb [14]. The induction of neocentromeres, as demonstrated in *S. pombe* and *Candida albicans* [15,16], has recently been applied in DT40 cells (Figure 1). Cre-induced deletion of 127 kb containing the Z centromere generated neocentromeres in 126 surviving colonies that retained the Z chromosome [17^••^]. This large number of novel neocentromeres can potentially be used to identify common features at these chromosomal locations that may promote CENP-A incorporation. Eighteen neocentromeres that formed on distinct Z chromosome sequences were further characterised. CENP-A peaks were confined to 35–47 kb regions, with no preference for the presence of repetitive elements. Most neocentromeres arose in regions flanking the original Z centromere, suggesting that a low level of CENP-A, resident in these regions at the time of centromere deletion, may seed new centromere formation. Examination of several induced neocentromeres in *Candida* also demonstrates that they frequently arise in close proximity to the original centromere [16,18]. Neocentromeres attract most centromere/kinetochore proteins (an exception being the satellite DNA-binding protein CENP-B) and allow efficient chromosome segregation. However, one human neocentromere was shown to inefficiently correct unsuitable spindle attachments, thus neocentromeres may not confer the same level of accuracy in segregation as natural centromeres [19].

Analyses in *S. pombe* and *Drosophila* indicate that heterochromatin influences the establishment of CENP-A chromatin and functional kinetochores [20–22]; however, H3K9 methylation, the key mark associated with heterochromatin, was not enriched at normal non-repetitive DT40 cell centromeres or induced neocentromeres. In *Caenorhabditis elegans*, CENP-A is deposited on chromosomal regions not transcribed in the germline and the lack of heterochromatin proteins (HP1) does not affect *de novo* centromere assembly on injected plasmids, suggesting that heterochromatin is not required [23,24]. Fission yeast heterochromatin acts as a platform to recruit many activities including several HDACs, chromatin remodelers, replication initiators and DNA repair proteins [25]. The concentration of associated activities may be responsible for promoting CENP-A^Cnp1^ rather than heterochromatin itself. Such activities may be found elsewhere on chromosomes, without standard heterochromatin features, and alone may be sufficient to promote CENP-A assembly. Further analyses of multiple neocentromeres, generated on the same genetic background, should illuminate how CENP-A assembly is triggered at new chromosomal locations.

## Preventing centromere formation

The formation of neocentromeres shows that the normal centromere locus on a chromosome is not unique in being able to attract CENP-A and assemble kinetochores. In addition, mechanisms exist that inactivate centromeres causing centromere protein loss, without affecting the DNA sequence itself, and that also prevent their reactivation [9]. Centromere inactivation has recently been shown to occur in fission yeast following the forced recombination between two non-homologous chromosomes to induce dicentric chromosome formation (Figure 1) [26^••^]. The dicentric state was deleterious but a proportion of surviving cells retained the dicentric chromosome with both centromere regions intact. However, CENP-A^Cnp1^ was found to be lost from either centromere, and retained at the other. The CENP-A^Cnp1^ negative centromere was not pulled to the spindle pole in anaphase, which is consistent with centromere inactivation. More survivors arose when dicentric formation was forced in cells with a defective kinetochore component, suggesting that kinetochore disassembly promotes centromere inactivation. Domains of H3K9me-dependent heterochromatin flank the central kinetochore domain at fission yeast centromeres. Heterochromatin was found to engulf the central domain at inactivated centromeres and prevent subsequent centromere reactivation. Surprisingly, however, heterochromatin itself is not required for centromere inactivation, but prevents the reactivation of dormant centromeres. Thus, when coated in heterochromatin, intact centromeric chromatin is epigenetically silenced and rendered unrecognisable so that it is unable to direct kinetochore assembly. The targeting of H3K9 methylation and heterochromatin to human α-satellite repeats also inhibits *de novo* CENP-A and kinetochore assembly [27]. However, heterochromatin also promotes *de novo* CENP-A and kinetochore assembly on naïve DNA templates in fission yeast [20,21]. The demarcation of heterochromatin domains relative to CENP-A domains, and their interplay, must influence whether nearby heterochromatin promotes or prevents CENP-A and kinetochore assembly. The details of how centromeres are inactivated and how heterochromatin prevents CENP-A deposition are currently unknown.

## Directing CENP-A and kinetochore assembly

**Figure 2:**
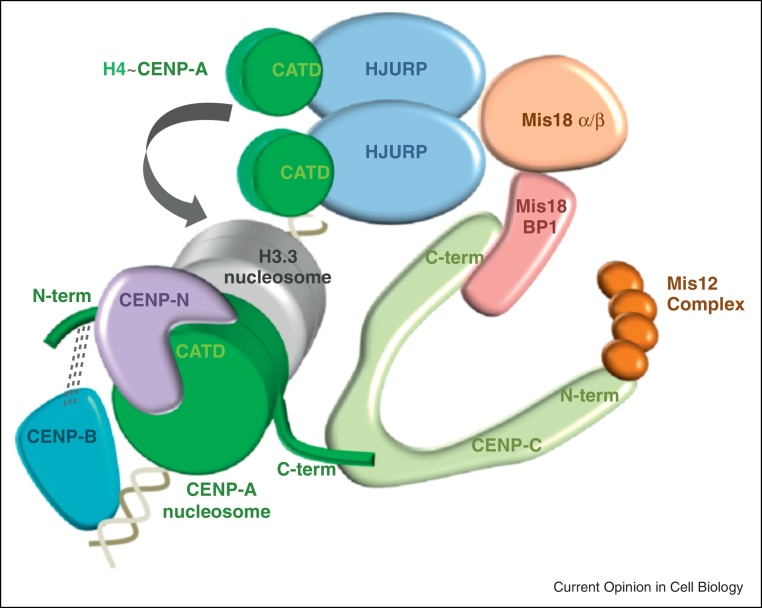
CENP-A recognition and propagation at regional centromeres. CENP-C binds directly to the C-terminus of CENP-A in nucleosomes. The C-terminus of CENP-C recruits the Mis18 complex through Mis18BP1 (also known as Knl2). During replication the CENP-A nucleosomes are distributed equally to each sister-centromere so that CENP-A levels are halved and histone H3.3 is deposited as a placeholder. Free CENP-A/H4 heterodimers associate with a homodimer of HJURP which is recruited to centromeres via the Mis18 complex in telophase, allowing replacement of H3.3 with CENP-A in G1. Once assembled, the CATD within the HFD of CENP-A nuclesomes is recognised by CENP-N allowing recruitment of many other constitutive kinetochore components including the CENP-T/W/S/X complex (not shown). The CENP-B protein is known to bind directly to centromere repeats in mammals but is stabilised via the N-terminus of CENP-A and contributes to kinetochore integrity. The N-terminus of CENP-C associates with the Mis12 complex (see Figure 3). For simplicity interactions and nomenclature are only shown for vertebrate proteins.

In vertebrate cells CENP-A is deposited at centromeres in early G1, independently of replication [28]. Deposition of CENP-A is dependent on its chaperone, HJURP [29,30]. HJURP associates with soluble CENP-A and is transiently recruited to centromeres in early G1. Recruitment of HJURP is dependent on the Mis18 Complex (Mis18α/β/Mis18BP1), which arrives at centromeres before HJURP in telophase [31^•^,32]. The centromere localisation of Mis18BP1 is inhibited by Cdk1/Cdk2-mediated phosphorylation from S phase to anaphase [33^•^]. CENP-C has been shown to discriminate CENP-A from H3 nucleosomes by binding directly to the distinct C-terminus of CENP-A and docking via acidic patches on histones H2A and H2B [34^•^,35]. CENP-N recognises CENP-A nucleosomes via the CENP-A targeting domain (CATD) within the histone-fold domain (HFD), even when transplanted into H3, and acts in conjunction with CENP-C to recruit other kinetochore components [36,37]. CENP-C directly binds Mis18BP1 and Mis18BP1/Mis18α/β is required to recruit HJURP to centromeres [32,38]. In fission yeast, Mis18 directly interacts with Scm3^HJURP^ [39]. Interactions such as these create a loop where resident CENP-A recruits the chaperones that mediate the deposition of new CENP-A (Figure 2). During S phase, resident CENP-A at human and *Drosophila* centromeres is distributed equally to sister-centromeres, each inherits half the amount originally present at the fully replenished parental centromere [28,40^•^]. Where CENP-A is lost, the resulting gaps are perhaps temporarily filled by deposition of H3.3 as a placeholder in S phase until its replacement with CENP-A in G1 [41]. HJURP has been shown to self-dimerise, which may enable it to deposit two new CENP-A/H4 heterodimers, thus allowing CENP-A nucleosome assembly in place of H3.3 nucleosomes in G1 [42].

Use of a human a cell line harbouring a conditional null allele of CENP-A demonstrated that one third of CENP-C was retained even when CENP-A was reduced to minimal levels (∼1%) and high levels of other kinetochore proteins persisted until CENP-A was undetectable [43^••^]. This suggests that kinetochore proteins can stabilise their own platform without CENP-A and only a small proportion of the normal amount of centromeric CENP-A is required to provide kinetochore function. These cells also allowed rigorous *in vivo* dissection of the distinct recognition modules within CENP-A and emphasise their two distinct roles in CENP-A propagation at a specific location and the assembly of kinetochores at that site. The CATD allows HJURP-mediated G1 deposition of CENP-A into, and its propagation in, distinct nucleosome particles. The N-termini and C-termini of CENP-A were found to be redundant with respect to promoting kinetochore assembly with CENP-C recruited by the C-terminus. CENP-B was shown to play a hitherto unrecognised role in kinetochore integrity by interactions via the N-terminus of CENP-A [43^••^]. This may relate to the finding that α-satellite DNA that binds CENP-B is a preferred substrate for *de novo* CENP-A and kinetochore assembly [10].

In *Drosophila* cultured cells, newly synthesised CENP-A^CID^ is incorporated at centromeres at metaphase [40^•^]. In the somatic tissues of flies, the incorporation of new CENP-A^CID^ at centromeres occurs from late telophase to early G1 [44^•^]. *Drosophila* lacks an HJURP ortholog but Cal1 performs the equivalent function and is recruited to centromeres via CENP-C [40^•^]. During meiosis, CENP-A^CID^ is replenished twice, once during prophase of MI and also in spermatids following the completion of MII. Both Cal1 and CENP-C are required for CENP-A^CID^ assembly at centromeres during meiosis. In mature *Drosophila* sperm most histones are replaced with protamines; however, as in vertebrates, CENP-A^CID^ is retained in sperm chromatin. The retention of CENP-A on sperm chromatin is required to ensure that kinetochores are assembled and that their location is preserved following fertilisation [44^•^,45^•^].

## Misguiding CENP-A assembly

To determine if CENP-A alone is sufficient to direct kinetochore assembly, CENP-A, or its chaperone HJURP, has been artificially tethered to DNA. In *Drosophila* S2 cells, tethering of LacI-CENP-A^CID^ to LacO arrays inserted on a chromosome arm recruits kinetochore components and mediates association with microtubule fibres [46^••^]. Untethered endogenous CENP-A^CID^ is also recruited to the tethering site and kinetochores persist after the initiating tethered LacI-CENP-A^CID^ is removed. Moreover, tethered CENP-A^CID^ conferred segregation function and mitotic stability to episomal plasmids. In human cells, tethering of HJURP to LacO sites also promoted the deposition of CENP-A and kinetochore assembly at an ectopic locus [31^•^]. These experiments demonstrate that CENP-A is indeed sufficient to specify centromeres and allow their propagation at that location.

Experimental overexpression of CENP-A may aid the identification of chromosomal features that promote its incorporation. Increased expression of CENP-A^CID^ in *Drosophila* cells showed that CENP-A^CID^ has a tendency to accumulate in proximity to heterochromatin [22]. In fission yeast, expression of additional CENP-A^Cnp1^ led to its accumulation close to heterochromatic telomeres, where neocentromeres are known to form [47^•^]. Telomere repeats themselves are sufficient to direct CENP-A^Cnp1^ incorporation nearby [48]. Defective transcription-coupled chromatin reassembly allows CENP-A^Cnp1^ accumulation on transcription units where H3 loss is greatest and also facilitates *de novo* deposition of CENP-A^Cnp1^ on fission yeast centromeric DNA, which is known to be transcribed [49]. Thus, alterations in the process of chromatin assembly during transcription can destabilise H3 nucleosomes and thereby allow CENP-A^Cnp1^ to assemble in its place [47^•^]. Interestingly, in mice with reduced levels of H3.3, which normally replaces canonical H3 in transcribed genes, CENP-A is deposited broadly over the genome [50]. Such findings suggest that transcription-coupled modification and remodelling events can influence the incorporation of CENP-A.

## CENP-A chromatin distinction

Canonical nucleosomes are octamers that contain two subunits each of the core histones H2A, H2B, H3 and H4. The substitution of H3 with CENP-A alone should be sufficient to allow recognition of these specialised nuclesomes. Indeed, structural analyses demonstrate that octameric CENP-A nucleosomes assembled *in vitro* are overall very similar to canonical H3 nucleosomes. One difference is that the αN-helical domain in CENP-A is shorter than that of H3, so less DNA is bound near the entry and exit sites [51^•^]. Consistent with this octameric structure, human centromeric CENP-A nucleosomes extracted from cells protect less DNA than H3 nucleosomes [52^•^]. AFM measurement of CENP-A particles extracted from *Drosophila* and human cells revealed that they have a reduced height relative to H3 nucleosomes and their height changes during the cell cycle [53,54,55^•^]. This height difference is central to the proposal that CENP-A particles are hemisomes (half-nucleosomes) rather than octamers. However, *in vitro* assembled octameric CENP-A nucleosomes also report a lower height relative to H3 nucleosomes [56^•^]. Thus, rather than indicating a different stoichiometry, the difference in height detected by AFM appears to be an intrinsic property of octameric CENP-A nucleosomes. Other analyses show that *Drosophila* CENP-A^CID^ can be cross-linked as dimers *in vivo*, demonstrating that CENP-A^CID^ nucleosomes contain two rather than one subunit of CENP-A^CID^ [57^•^]. Moreover, counting the number of CENP-A signals in single nucleosomes released from human cellular chromatin using TIRF revealed the presence of two CENP-A subunits in the majority of particles [58^•^]. Thus the major difference in composition between CENP-A and H3 nucleosomes appears to be the replacement of both H3 subunits with CENP-A. As discussed above, the N-terminal and C-terminal regions, along with the CATD, mediate specific interactions that distinguish CENP-A from H3 nucleosomes.

## Post-translational modifications on CENP-A

Histones are subject to a slew of PTMs that regulate the binding of specific proteins. Unlike H3, the N-terminal region of CENP-A is highly variable in sequence and length. Several CENP-A PTMs have been identified. *S. cerevisae* CENP-A^Cse4^ was shown to be methylated on Arg37, acetylated on Lys49 and phosphorylated on Ser22, Ser33, Ser40 and Ser105 [59]. An Arg37Ala mutation in CENP-A^Cse4^ resulted in reduced association of specific kinetochore components with centromeres [60]. Phosphorylation is mediated by Ipl1, the Aurora B kinase, and mutation of all 4 residues suggests a role for CENP-A^Cse4^ phosphorylation in regulating sister-kinetochore bi-orientation [59]. Phosphorylation of human CENP-A on Ser7 by Aurora kinase plays a role in kinetochore function and cytokinesis [61]. Recent analyses suggest that any phosphorylation within the N-terminus of CENP-A (S7 of CENP-A or S10/S28 from H3 N-termini are sufficient) is required during mitosis to stabilise the CENP-C recruitment via phospho-binding 14-3-3 proteins [62]. Like other histones, the initiating methionine of human CENP-A is removed post-translationally, so that Gly1 is the first residue in nucleosomal CENP-A and is trimethylated on its primary amine [63^•^]. Simultaneous phosphorylation of both Ser16 and Ser18 was also detected at high levels on peptides that carried the Gly1me3 modification. The phosphorylation of Ser16 and Ser18 appears to affect kinetochore integrity. How this phosphorylation relates to Serine-7 phosphorylation and 14-3-3 recruitment is not known.

## Other histone-related proteins at centromeres

Apart from CENP-A, four conserved histone-fold fold proteins (CENP-T/-W/-S/-X known as Cnn1/Wip1/Mhf1/Mhf2 in *S. cerevisiae*) have been shown to reside at vertebrate centromeres. CENP-T interacts directly with CENP-W and together these four proteins form a CENP-T/W/S/X heterotetrameric complex via their HFDs that assembles on DNA in a manner reminiscent of histone tetramers [64^•^]. Analyses of specific disruptive mutants in DT40 cells demonstrate that the formation of this tetramer is required to allow kinetochore assembly. CENP-T/W/S/X is therefore a second DNA binding module that operates alongside CENP-A to recruit kinetochore components. CENP-S and CENP-X are also known to associate with FANCM, a helicase that blocks cross-over formation as a result of homologous recombination by reversing D-loops [65,66]. FANCM might also be recruited to centromeres by CENP-S/-X where it could suppress potentially deleterious recombination events at centromeres, particularly those involving repetitive elements.

## Bypassing CENP-A

**Figure 3:**
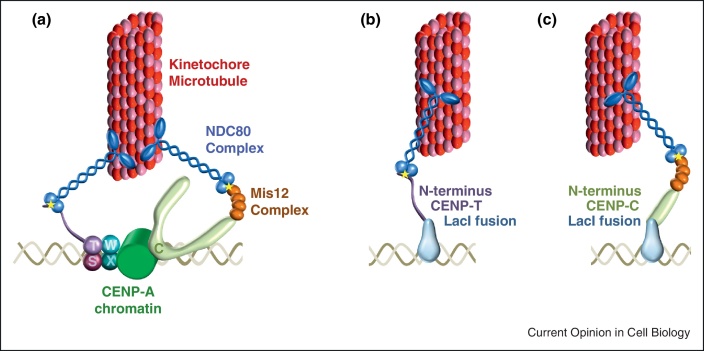
Two pathways connect centromere DNA to kinetochore microtubules via CENP-A. **(a)** The four HFD proteins in the CENP-T/W/S/X complex form a heterotetramer that is recruited via other constitutive kinetochore components, such as CENP-N that binds CENP-A nucleosomes (not shown — see Figure 2). An extended structure in CENP-T separates the HFD near the C-terminus from the N-terminus. The N-terminus of CENP-T contains a short motif that directly associates with the RWD motif formed by Spc24/Spc25 of the NDC80 complex (star). CENP-C binds CENP-A nucleosomes and its N-terminus recruits the Mis12 complex. The Nsl1 subunit of the Mis12 complex directly associates with the RWD motif formed by Spc24/Spc25 of the NDC80 complex (star). The opposite end of NDC80 complex binds directly to microtubules. Fusion of the N-terminus of CENP-T **(b)**, or the N-terminus of CENP-C **(c)**, to LacI allows their artificial recruitment to arrays of LacO (LacI binding sites) at a non-centromeric locus where they can connect with microtubules and mediated chromosome segregation in the absence of CENP-A nucleosomes. The N-terminus of Cnn1, the *S. cerevisiae* ortholog of CENP-T, is also sufficient to mediate chromosomes segregation when tethered to plasmid DNA. For simplicity interactions and nomenclature are only shown for vertebrate proteins.

Kinetochores are essentially an elaborate ‘towbar’ that mediates and regulates the connection between microtubules and chromosomal DNA. Thus, the means of connecting microtubules to DNA may not be particularly important; provided the modules that bind DNA are linked to modules that can attach to microtubules, perhaps chromosome segregation can occur. A conserved N-terminal motif, several hundred residues from the HFD of CENP-T/Cnn1, directly associates with the Spc24/25 end of the NDC80 microtubule binding complex (Figure 3) [67^•^,68^•^]. Direct tethering of TetR-Cnn1 or LacI-CENP-T fusion proteins to TetO/LacO arrays can mediate chromosome segregation without CENP-A in human, DT40 and *S. cerevisiae* cells, but not when this NDC80 interaction motif is deleted [69^••^,70^••^]. Thus this N-terminal motif in CENP-T/Cnn1 plays a conserved role in connecting DNA to microtubules via the NDC80 complex. The NDC80 complex is also anchored at kinetochores through CENP-A via CENP-C and its interaction with the Mis12 complex (Mis12/Nnf1/Dsn1/Nsl1/) [71]. The Nsl1 component directly interacts with Spc24/25 of the NDC80 complex to allow kinetochore–microtubule interactions [72]. Consequently, tethering the N-terminal region of CENP-C (which associates directly the Mis12 complex, but does not recruit CENP-A) to LacO arrays also allows chromosome segregation, in the absence of CENP-A, in DT40 cells [69^••^]. Interestingly, *Drosophila* and *C. elegans* lack CENP-T along with other kinetochore proteins [73] and thus rely on the CENP-C/Mis12/Ndc80 pathway to connect with microtubules [74].

The tethering experiments discussed above demonstrate that the need for CENP-A chromatin can be bypassed when an alternative way of connecting kinetochore components to DNA is provided. It is therefore perhaps surprising that CENP-A has persisted through evolution as the major DNA binding unit at centromeres in eukaryotes. The continued use of a system that allows the epigenetic regulation of CENP-A deposition and plasticity in the placement of centromeres may have an important evolutionary role in permitting the survival of novel chromosome arrangements that ultimately may drive speciation. For organisms with genetically determined, DNA sequence dependent, centromeres, it is possible that the wrapping of centromeric DNA around specialised nucleosomes is required to resist the pulling forces exerted on centromeres during chromosome segregation and consequently CENP-A has been preserved. Intriguingly, so far no CENP-A related protein is evident within the sequenced genomes of kinetoplastids such as Trypanasoma, which occupy an ancient evolutionary niche [75]. Perhaps nature has also found ways of building kinetochores that do not involve CENP-A or chromatin and that connect with DNA more directly.

## Conclusion

The identification of components that recognise different parts of CENP-A in nucleosomes, and consequently lead to the assembly of functional kinetochores has identified pivotal connections between CENP-A and constitutive kinetochore proteins. Tethering experiments have allowed the identification and dissection of components that link centromeric DNA with microtubules. It remains to be determined exactly how specific loading factors mediate the replication-independent deposition of CENP-A. The development of systems that promote neocentromere formation and centromere inactivation will continue to provide insights into the mechanisms that activate and repress centromeres. The features in chromatin that initiate *de novo* CENP-A deposition at specific chromosomal locations have yet to be identified.

“You need chaos in your soul to give birth to a dancing star” — Friedrich Nietzsche

## References and recommended reading

Papers of particular interest, published within the period of review, have been highlighted as:

• of special interest
•• of outstanding interest
